# Urinary Retention as an Initial Clinical Manifestation of Primary Sjögren's Syndrome

**DOI:** 10.7759/cureus.72729

**Published:** 2024-10-30

**Authors:** Bshara Sleem, Victor Ghazi, Georges El Hasbani, Imad Uthman

**Affiliations:** 1 Orthopedic Surgery, American University of Beirut, Beirut, LBN; 2 Internal Medicine, American University of Beirut, Beirut, LBN; 3 Rheumatology, Mayo Clinic, Rochester, USA; 4 Rheumatology, American University of Beirut, Beirut, LBN

**Keywords:** autoimmunity, imaging, serology, sjogren's syndrome, urinary retention

## Abstract

Primary Sjögren's syndrome (pSS) is a systemic autoimmune disease known to affect exocrine glands, leading to symptoms such as dry eyes and mouth. However, pSS can manifest in other systems, including rare genitourinary presentations. Urinary retention, although unusual, has been associated with pSS throughout the disease, particularly when related to neurogenic bladder dysfunction. However, it is unusual for neurogenic bladder dysfunction to be the initial presentation of pSS. We present the case of a 38-year-old woman who initially presented with unexplained urinary retention, followed by the development of systemic symptoms such as hand arthritis, morning stiffness, photosensitivity, and dry eyes. As Shirmer's test was positive and anti-SSA was strongly positive, she was diagnosed with pSS. Treatment with hydroxychloroquine alleviated both her systemic symptoms and urinary retention.

## Introduction

Primary Sjögren's syndrome (pSS) is a systemic inflammatory autoimmune disease that primarily affects the lacrimal and salivary glands but can also involve the musculoskeletal, hepatic, renal, pulmonary, and neurologic systems [1]. Less commonly, genitourinary symptoms have been associated with pSS. For example, bladder pain syndrome has been reported in 10.6 cases per 105 patients per year [2]. Overactive bladder has also been reported as a manifestation of pSS in around 50% of the cohort. The proposed mechanism for such an association involves binding to the M3 muscarinic receptor (M3R), causing exocrine dysfunction and leading to bladder detrusor smooth muscle contraction [3].

Urinary retention is one of the common manifestations of multiple diseases. For example, a urinary tract infection or cholinergic toxicity might cause urinary retention [4]. The association between urinary retention and pSS has been rarely reported and is mainly linked to spinal cord pathologies or medication side effects [5,6].

Herein, we present a case of a young female who initially presented with unexplained urinary retention and developed sicca symptoms and arthritis a few months later in the setting of positive anti-SSA. Treatment with hydroxychloroquine helped alleviate the urinary symptoms in addition to the pSS symptoms.

## Case presentation

**Figure 1:**
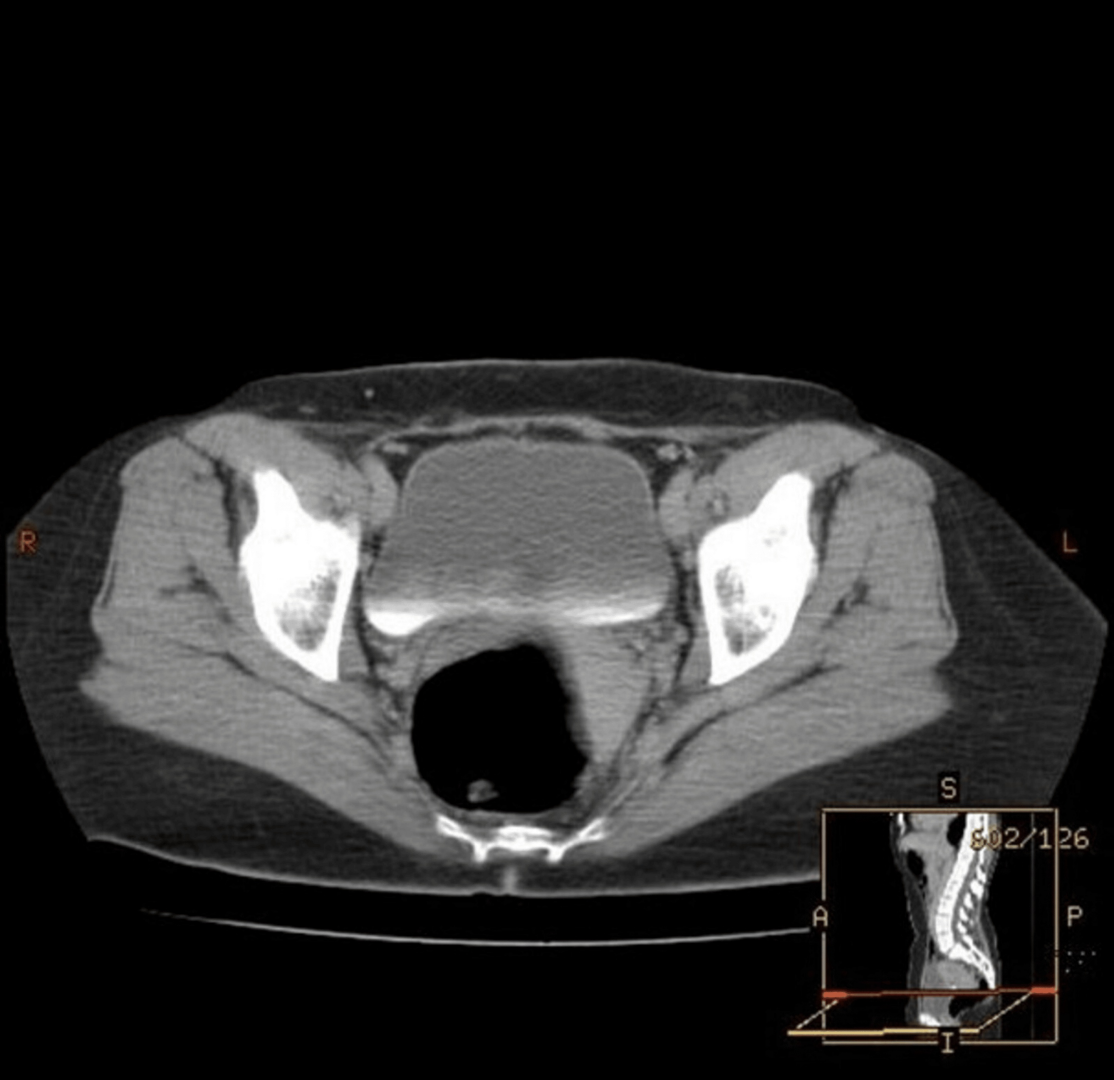
CT of the pelvis (axial view) demonstrated normal bladder with no signs of inflammation.

A 38-year-old previously healthy female presented to the emergency department with a three-day history of flank pain and urinary retention. She reported increased abdominal pressure and an urge to void, but inability to do so. She denied increased urinary frequency prior to the retention. She also denied loss of sensation in her lower extremities. Blood and urine investigations were non-revealing for an infectious etiology. Pelvic imaging by CT scan and MRI was also non-revealing for an acute pathology (Figure 1).

As such, myocholine and tamsulosin were started, and a Foley catheter was inserted, yielding 1.6 L of clear urine. She was discharged from the hospital with a Foley catheter. She failed voiding trial twice. 

Five months later, she presented to the emergency department with hemoptysis. She also reported new-onset hand arthritis, morning stiffness, photosensitivity, Raynaud's phenomenon, and dryness of the eyes. The patient had a history of three first-term miscarriages. There was no evidence of parotid gland enlargement, nasopharyngeal/oral ulcers, or vaginal dryness on physical examination. Schirmer strip test was positive at 8 mm. Laboratory investigations were significant for slightly elevated C-reactive protein (6 mg/dL) and highly elevated anti-SSA (Ro) and anti-Ro-52 antibodies (Table 1).

**Table 1:** A complete autoantibody profile.

Autoantibodies	Level of Detection
Mi-2	-
Ku	-
RNP/Sm	-
Sm	-
SS-A native (60 kDa)	++
Ro-52 recombinant	+
SS-B	-
Scl-70	-
PM/Scl-100	-
Jo-1	-
Centromere B	-
PCNA	-
dsDNA	-
Nucleosomes	-
Histones	-
Ribosomal protein	-
AMA-M2	-
DFS70	-
Antinuclear	-
Lupus anticoagulant	-
Beta-2 glycoprotein 1	-
Anti-cardiolipin	-

A diagnosis of Sjogren's syndrome was made. She was started on hydroxychloroquine 400 mg daily. A follow-up eight weeks later demonstrated an improvement in her arthritis and morning stiffness. Additionally, a voiding trial was performed, and the patient was able to void spontaneously.

## Discussion

One of the most common genitourinary manifestations in women with pSS is vaginal dryness, which results from reduced glandular secretions and can lead to dyspareunia, increased risk of vaginal infections, and overall discomfort [7,8]. Urinary tract manifestations are also seen in pSS, although less frequently reported. Interstitial cystitis, characterized by chronic bladder pain and urinary urgency without an underlying infection, is one such manifestation [9]. Patients may experience urinary frequency, like symptoms seen in other forms of chronic cystitis [10]. The pathophysiological process is thought to involve inflammation of the bladder wall caused by autoimmune activity, similar to how other glands in the body are affected [11]. Another significant genitourinary manifestation in pSS is renal tubular acidosis, resulting from autoimmune tubulointerstitial nephritis (TIN) [12].

Urinary retention, though rare, is a notable manifestation that may arise due to neurogenic bladder dysfunction [13]. SS patients are found to have serum auto-antibodies that target muscarinic M3 receptors (M3R) on the detrusor smooth muscle and antagonize its normal contraction response [14,15]. Thus, in addition to the classical characterization of exocrine gland dysfunction secondary to inflammation and lymphocytic infiltration, a more complete characterization of pSS would entail receptor-mediated humoral auto-immunity, similar to what can be seen in other autoimmune conditions like Graves' disease and myasthenia gravis [14]. Moreover, while some data positively correlate anti-M3R auto-antibodies with anti-SSA/Ro antibodies [16,17], other data find no such correlation [18]. There have been previously reported cases of urinary retention in the setting of Sjogren's syndrome, and they are summarized in Table 2.

**Table 2:** Characteristics of case reports that discussed urinary retention in the setting of primary Sjögren's syndrome.

Author (Year)	Age/ Sex	Symptoms	Diagnosis	Treatment	Outcome
Caretti et al. (2023) [19]	72/F	Nausea, vomiting, and urinary retention	Primary Sjögren's syndrome with distal renal tubular acidosis	Intravenous fluids, antibiotics, and sodium bicarbonate	Complete Recovery
Menor Almagro et al. (2015) [20]	34/F	Optic neuritis and urinary retention	Sjögren's syndrome with transverse myelitis	Glucocorticoids and azathioprine	Complete Recovery
Verma et al. (2013) [21]	40/F	Urinary retention and incomplete emptying of the bladder	Sjögren's syndrome with acute myeloneuropathy	Glucocorticoids	Improved muscle power, but persistent urinary incontinence
Choi et al. (2006) [22]	43/F	Paraparesis, sensory abnormalities, urinary retention	Primary Sjögren's syndrome with transverse myelitis	Glucocorticoids and cyclophosphamide (750 mg) every 4 weeks	Complete recovery
Sorajja et al. (1999) [23]	51/F	Urinary retention and esophageal dysmotility	Primary Sjögren's syndrome	Prednisone and hydroxychloroquine	Complete recovery
Niemelä et al. (1999) [24]	24/F	Personality change, confusion, seizures, and neurogenic bladder	Primary Sjögren's syndrome with neurogenic bladder	Glucocorticoids, cyclophosphamide, and carbamazepine	Refractory

However, it is important to recognize that when bladder symptoms do arise in pSS patients, they frequently result from bladder and detrusor muscle overactivity, rather than underactivity. A proposed explanation for this paradox is that the initial inhibition of cholinergic stimulation by anti-M3R auto-antibodies is likely followed by a compensatory overexpression of M3R [25], a resultant increased sensitivity to acetylcholine during bladder distention, and subsequent involuntary detrusor muscle contractions or overactivity [18,26]. Animal studies have verified the compensatory increase in M3R expression, and subsequent cholinergic hyperresponsiveness, by immunofluorescence examination of the bladders of mice injected with pSS and secondary Sjögren's syndrome patients' IgG antibodies [18].

The management of neurogenic dysautonomia secondary to pSS is a complex and multifaceted process. The European Alliance of Associations for Rheumatology (EULAR) Sjögren's syndrome disease activity index (ESSDAI) addresses several systemic manifestations of pSS, but it fails to capture neurogenic dysautonomia when it pertains to the bladder [27]. In recent years, intravenous immunoglobulin (IVIg) has emerged as a potential therapeutic option for patients with pSS-related neurogenic dysautonomia [28-30]. IVIg has been shown to be particularly effective in patients with pSS who exhibit significant autonomic nervous system involvement. The administration of IVIg can lead to improvement in autonomic symptoms such as orthostatic hypotension, gastroparesis, and neurogenic bladder dysfunction [31]. This improvement is thought to result from the immunomodulatory effects of IVIg, which can reduce inflammation and potentially restore some degree of autonomic nerve function.

In addition to IVIg, the management of neurogenic dysautonomia in pSS may include other pharmacological treatments aimed at specific symptoms. For example, patients with orthostatic hypotension might benefit from fludrocortisone or midodrine [32], while those with neurogenic bladder dysfunction may require anticholinergic medications or intermittent catheterization to manage urinary retention and incontinence [33]. Non-pharmacological interventions, such as dietary modifications and physical therapy, can also play a role in symptom management [34].

Our case is unique in that urinary retention preceded the systemic manifestations of pSS and was the initial clinical manifestation of pSS. Notably, the treatment of pSS with hydroxychloroquine led to the resolution of urinary retention.

## Conclusions

Primary Sjögrenäs syndrome (pSS) is characterized by numerous extra-articular manifestations, including genitourinary symptoms. Although pSS has been associated with urinary retention, it is rare for urinary retention to be the initial manifestation, as observed in our case. Interestingly, controlling disease activity, with disease+modifying agents such as hydroxychloroquine, may lead to a control of the genitourinary symptoms. 
